# Scimitar syndrome: report of a case and its surgical management

**DOI:** 10.4103/0256-4947.51820

**Published:** 2009

**Authors:** Ahmad Rajaii-Khorasani, Mahdi Kahrom, Hassan Mottaghi, Hadi Kahrom

**Affiliations:** a From the Division of Cardiothoracic Surgery, Ghaem & Razavi Hospital, Mashhad University of Medical Sciences, Mashhad, Iran; b From the Division of Cardiology, Department of Pediatrics, Imam Reza Hospital, Mashhad University of Medical Sciences, Mashhad, Iran

Scimitar syndrome is a rare condition consisting of anomalous pulmonary venous drainage from the right lung to the inferior vena cava (IVC). The extent to which the right lung is drained by the scimitar vein varies from partial to complete. Scimitar syndrome has been associated with a number of other abnormalities including hypoplastic right lung, anomalous systemic arterial supply to the right lung with or without pulmonary sequestration, pulmonary hypertension, dextroposition of the heart and atrial septal defect (ASD), with ostium secundum being the most common.^1^ The term scimitar syndrome derives from the shadow created by the anomalous vein on the chest radiograph. This shadow extends from the lateral superior position of the right lung to a more medial location and increases in caliber as it descends toward the cardiophrenic angle. The appearance closely resembles that of a curved Turkish sword or scimitar.^2^ This rare anomaly has an incidence of approximately 1 to 3 per 100 000 live births;^3^ the true incidence may be higher because many patients are asymptomatic.

## CASE

A 21-year-old female college student was admitted to the Department of Surgery with a mesenteric cyst. After performing a chest x-ray, she was referred to the Department of Cardiology because of an incidentally detected dextrocardia. She denied shortness of breath, paroxysmal nocturnal dyspnea, orthopnea, wheezing, fever, cough or any known cardiopulmonary disease. She had no history of syncope, lightheadness or diaphoresis. Her family history was unremarkable and specifically negative for cardiopulmonary anomalies. Of note, her parents reported a history of recurrent respiratory infections in her childhood.

Physical examination revealed a well-appearing youth in no apparent distress. Chest examination demonstrated normal chest wall excursion and clear breath sounds bilaterally. The heart was regular in rate; S1 was normal and S2 was widely split and did not vary with respiration.

**Figure 1:**
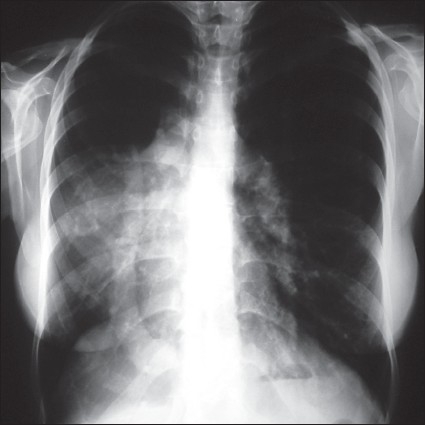
Posterior-anterior x-ray of the chest. Note that the heart is shifted into the right chest. The right lung is hypoplastic. Also note the “scimitar sign”-a curvilinear opacity widening in its course to the inferior vena cava.

**Figure 2:**
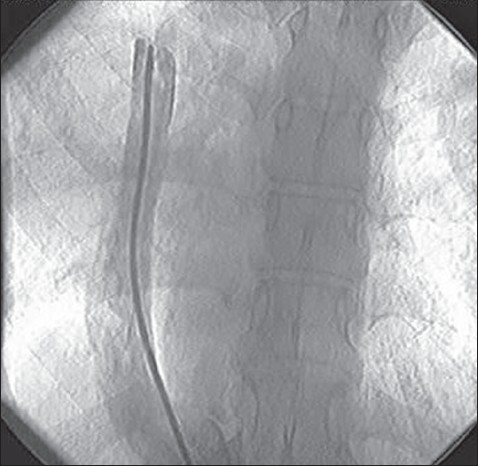
Cardiac catheterization displaying the anomalous right pulmonary vein.

Heart sounds were shifted to the right with a grade 2/6 ejection murmur. The chest radiograph showed an abnormal shadow arising from the inferior margin of the right lung hilum and dextroposition of the heart, suspicious of scimitar syndrome (Figure 1). Two-dimensional and Doppler echocardiography demonstrated marked ASD and true tricuspid regurgitation. Cardiac catheterization and angiography displayed the aberrant vein inserting to the IVC (Figure 2) and hypoplasia of the right pulmonary artery with no abnormal arterial supply to the right lung from the aorta. Oxygen saturation of the IVC and right atrium were 98.7% and 88.3%, respectively. Systolic pulmonary artery pressure was 55 mm Hg. Pulmonary hypertension in combination with a large ASD made our patient a candidate for surgical repair.

### Operative technique

**Figure 3:**
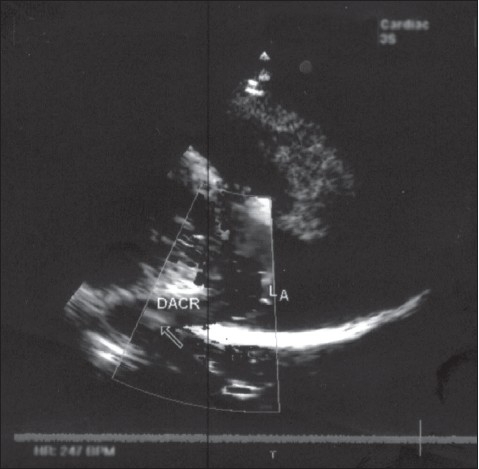
Trans-esophageal echocardiography showing the patent Dacron graft draining into the left atrium.

At the time of surgery, under deep hypothermic circulatory arrest, the right atrium and the IVC were opened. The end of the scimitar vein on the diaphragm was suture ligated. A 12-mm hemoshield graft was anastomosed end to side, to a longitudinal venotomy. The other end of the graft was brought through an opening in the back of the left atrium and the end was sewn from inside to the full thickness of the wall of the left atrium. The ASD was then closed with a pericardial patch. The right atrium was closed and the circulation was reestablished. At 4 years, there were no post-operative complications. Subsequent trans-esophageal echocardiography demonstrated a well-functioning graft (Figure 3). She has been treated with warfarin and aspirin.

## DISCUSSION

Functionally, scimitar syndrome resembles an ASD. It occurs more commonly in females and is occasionally familial. The left lung is very rarely involved for unknown reasons. The indications for surgical repair include the presence of scimitar syndrome, especially in association with ASD, pulmonary hypertension or stenosis of the anomalous vein. Various operative approaches are used to correct scimitar syndrome. The first report of surgical therapy for scimitar syndrome was in 1950 by Drake and Lynch, who performed a right lower lobectomy.^4^ To avoid lung resection and complications associated with simple ligation of the anomalous pulmonary vein, Kirklin et al^5^ in 1956 tried the first total correction without cardiopulmonary bypass. Several methods with cardiopulmonary bypass have been recommended to repair this anomaly, including direct anastomosis of the scimitar vein to the left atrium, as reported by Honey,^6^ or division with reimplantation of the anomalous pulmonary vein into the right atrium, as proposed by Shumacker and Judd.^7^ Alternatively, an intra-atrial patch may be used to create a tunnel, redirecting flow from the anomalous pulmonary vein to the left atrium through an ASD, as described by Zubiate and Kay in 1962.^8^ Puig-Massana and Revuelta^9^ described use of the free wall of the right atrium to create a tunnel from the scimitar vein to the left atrium across an ASD. There have been two case reports utilizing a 14-mm Dacron graft^10^ and a 20-mm Dacron graft^11^ interposed between the orifice of the anomalous pulmonary vein and an enlarged ASD as an intra-atrial conduit.

During the surgery, we could not identify the entrance of the scimitar vein into the atriocaval junction. We believe that in this case, the scimitar vein entered the hepatic venous system. Furthermore, the scimitar vein was crossing the diaphragm near its dome and was not long enough to be directly transferred or easily released because of inserting venous collaterals. The use of a Dacron graft as an extra-cardiac conduit between the scimitar vein and the left atrium has not been previously reported. Although the fate of a Dacron graft in the venous position and the long-term patency is not clear, the graft in this case is patent and functioning well after 4 years. The use of an anticoagulant and aspirin (despite patient's preference to not take these drugs) was an attempt to maximize the graft patency.
